# Response of Resistant and Susceptible Bayberry Cultivars to Infection of Twig Blight Pathogen by Histological Observation and Gibberellin Related Genes Expression

**DOI:** 10.3390/pathogens10040402

**Published:** 2021-03-29

**Authors:** Haiying Ren, Yangchun Wu, Temoor Ahmed, Xingjiang Qi, Bin Li

**Affiliations:** 1 The Institute of Horticulture Zhejiang Academy of Agricultural Sciences, Hangzhou 310021, China; renhy@zaas.ac.cn (H.R.); qixj@zaas.ac.cn (X.Q.); 2 College of Chemistry and Life Science, Zhejiang Normal University, Jinhua 321004, China; wuyangchun1022@163.com; 3 State Key Laboratory of Rice Biology and Ministry of Agriculture Key Lab of Molecular Biology of Crop Pathogens and Insects, Institute of Biotechnology, Zhejiang University, Hangzhou 310058, China; temoorahmed@zju.edu.cn

**Keywords:** bayberry twig blight, *Pestalotiopsis*, invasion mechanism, gibberellin, gene expression

## Abstract

Bayberry is an important fruit tree native to the subtropical regions of China. However, a systematic twig blight disease caused by *Pestalotiopsis versicolor* and *P. microspora*, resulted in the death of the whole tree of bayberry. The main variety Dongkui is highly sensitive to the twig blight disease, but the variety Zaojia is very highly resistant to the disease. Therefore, it is very necessary to clear the difference between resistant and susceptible varieties in response to the fungal infection. In this paper, we investigated the response of resistant and susceptible bayberry cultivars to infection of twig blight pathogen by histological observation and gibberellin signaling pathway-related genes expression. Microscopic observation revealed the difference in the infection process between resistant and susceptible varieties. The results of frozen scanning electron microscopy showed that the *Pestalotiopsis* conidia were shrunk, the mycelium was shriveled and did not extend into the cells of resistant cultivars, while the conidia were full and the top was extended, the mycelia was normal and continued to extend to the cells of a susceptible cultivar. Indeed, the medulla cells were almost intact in resistant cultivar, but obviously damaged in susceptible cultivar after inoculation of the main fungal pathogen *P. versicolor* conidia, which is earlier germinated on sterile glass slide than that of a hard plastic slide. The quantitative real-time PCR results showed a significant difference between resistant and susceptible cultivars in the expression of gibberellin signaling pathway-related genes in leaves and stems of bayberry, which is closely related to infection time, the type of genes and varieties. Overall, this study provides a clue for our understanding of the resistance mechanism of bayberry against the twig blight disease.

## 1. Introduction

Bayberry (*Myrica rubra*) is an important subtropical fruit in China with unique flavor, high nutritional value and economic benefits, and enjoys a high reputation at home and abroad [1,2]. At present, the planting area of *Myrica rubra* in China is 333,300 hectares large and in Zhejiang Province is as high as 86,700 hectares, ranking the first in China. Because of its remarkable economic and ecological benefits, it has become the main fruit in Zhejiang Province, and its output value has steadily ranked the first in all kinds of fruits in Zhejiang Province. In 2004, a systematic twig blight disease was first found in Xianju County, Zhejiang Province. Until 2007–2008, this disease suddenly broke out on a large scale, and the trees that infected for 3–4 years began to die. Nowadays, the disease has been found in the main producing areas (such as Ruian, Huangyan, Linhai, Qingtian) of bayberry in Zhejiang Province and many other provinces, including Fujian, Jiangxi, Guangxi, Guangdong and other places [3,4]. The outbreak of twig blight seriously restricted the development of the bayberry industry [5].

The twig blight disease is caused by *Pestalotiopsis versicolor* and *P. microspora*, while the former is the main pathogen [3]. The pathogen *Pestalotiopsis* spp. of bayberry twig blight belongs to Ascomycota, which has been reported to be an endophytic fungus and an important plant pathogen. The pathogen mainly colonizes in the phloem of branches and can cause wilt and leaf spots of bayberry [3,4,6]. In addition, the *Pestalotiopsis* spp. can cause fruit rot symptoms in guava [7], wither and canker symptoms in the top tip of blueberry [8], rot the stem tip of avocado [9], and gray leaf spot symptoms in mango [10], leading to branch blight, leaf spot, fruit rot and postharvest rot of Loquat [11,12]. However, the pathogenic mechanism of this pathogen to bayberry is complex [13].

Although some studies have found that the application of chemical fungicides such as difenoconazole, prochloraz, iprodione, pyrazoxystrobin and propiconazole can have a certain effect on the control of bayberry twig blight disease, the disadvantage of environmental pollution is also obvious [14,15]. Biological control of *P. versicolor* is still limited to the laboratory and has not been widely used in orchards [16,17]. In order to effectively control this disease, an alternate method is to popularize and cultivate resistant varieties. At present, compared with the highly susceptible cultivar Dongkui, Zaojia is the main variety of bayberry with high resistance to twig blight in the market. This resistance may be mainly due to its characteristics of dwarf trees and short fruit maturity [18]. However, up to now, there is no systematic study on the difference in the infection process between resistant and susceptible varieties of bayberry [6].

A lot of studies have shown that plant hormones play a key role in plant response to biotic and abiotic stresses [19,20,21]. Although salicylic acid and jasmonic acid pathways have been well elucidated in many previous studies, the signaling pathways of plant hormones are in general complex and diverse [22,23]. In particular, more and more recent studies indicated that gibberellin signaling may be involved in the resistance of the plant to adverse environments by crosstalking with jasmonic acid and salicylic acid signaling often accompanied by changes in plant size [24,25]. Therefore, a clearer understanding of the role of gibberellin signaling in the response of the bayberry plant to the pathogen infection would be an important step towards understanding and improving plant growth under adverse environmental conditions.

The aim of this study was to analyze the infecting process of this pathogen in bayberry, the response of resistant and susceptible cultivars to infection of twig blight pathogen was investigated by histological observation of the phloem, xylem and medulla cells as well as *P. versicolor* conidia and mycelial. Furthermore, the role of gibberellin signaling pathway-related genes in response of bayberry to pathogen infection was determined by comparing their expression in leaves and branches at different times after inoculation between resistant and susceptible cultivars.

## 2. Results

### 2.1. Conidia Germination and Morphology

**Figure 1:**
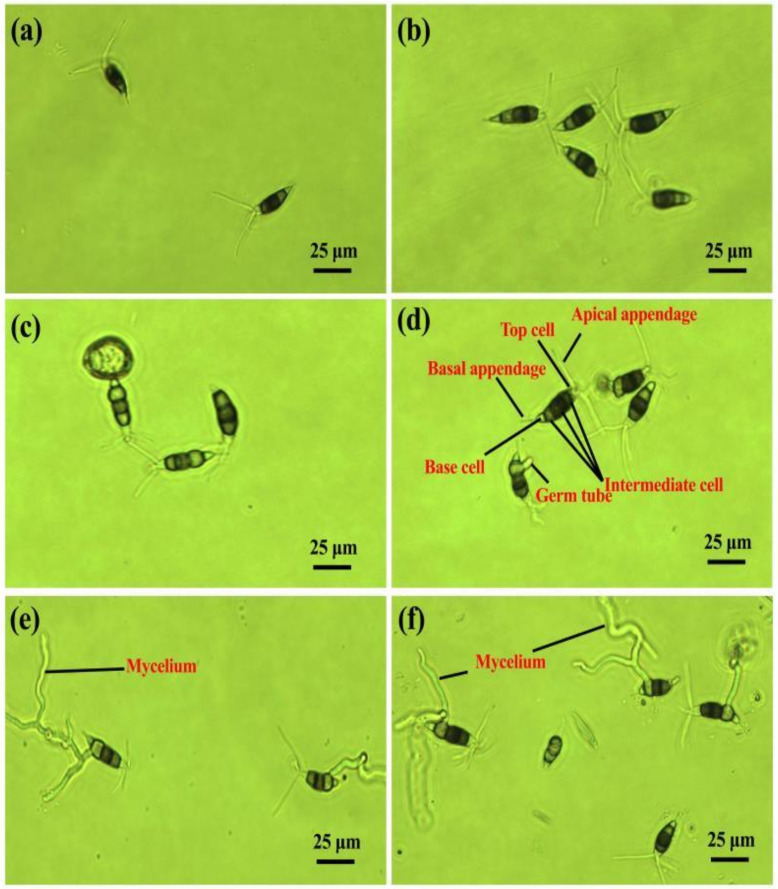
Germination of conidia of *P. versicolor* strain XJ27 on a glass slide and hard plastic. (**a**,**c**,**e**) glass slide at 0, 8 and 20 h of incubation (**b**,**d**,**f**) Hard plastic at 0, 10 and 20 h of incubation.

As shown in Figure 1, the width of conidia ranges from 4.95 to 8.82 μm, the length ranges from 20.69 to 28.98 μm; the length of basal appendages ranged from 2.78 to 6.82 μm, the length of apical appendages ranged from 11.89 to 27.18 μm, and the length of intermediate cells ranged from 13.27 to 18.32 μm. The middle cells of conidia of *P. versicolor* strain XJ27 are heterochromatic, the top and base cells are transparent and colorless; the bottom cell is the lightest, the shape is fusiform or spindle, the top attached 2–4 appendages, the base attached one appendage; the middle three cells are brown, the second cell is the darkest. Furthermore, the conidia germination of *P. versicolor* strain XJ27 was different on a glass slide and hard plastic. On the glass slide, the conidia began to germinate after 8 h of incubation, the penultimate cell at the base started to expand, and then the germ tube grew from the side of the cell. After 20 h of incubation, the germ tube further elongated and bifurcated and gradually formed hyphae. However, on hard plastics, the conidia germinated slowly and germinated after 10 h of incubation, which was similar to that on the glass slide.

**Figure 2:**
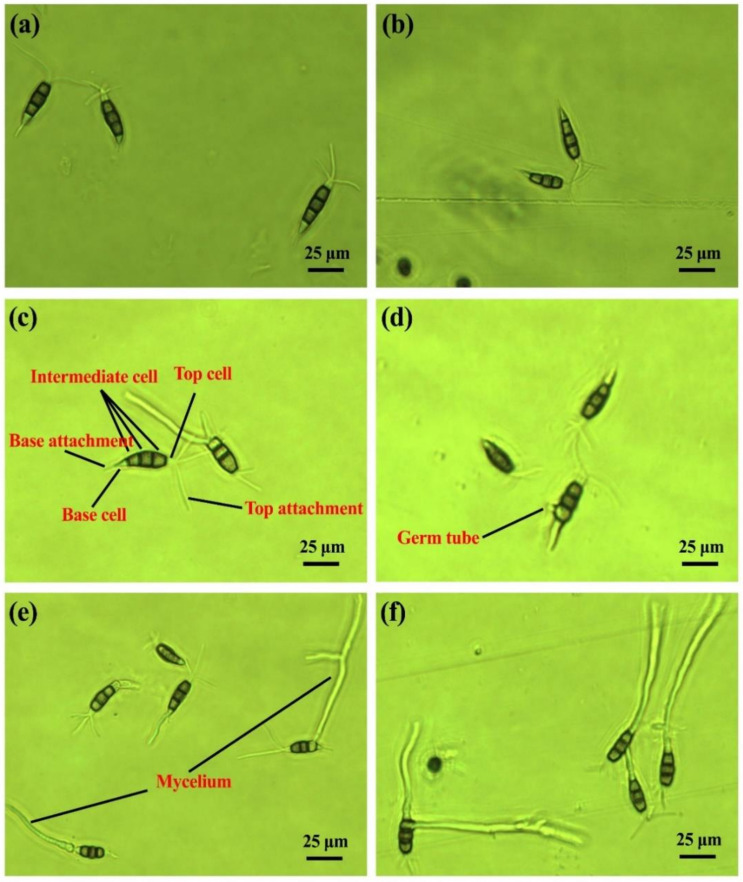
Germination of conidia of *P. microspore* strain YS26 on slide and hard plastic. (**a**,**c**,**e**) glass slide at 0, 10 and 20 h of incubation (**b**,**d**,**f**) hard plastic at 0, 10 and 20 h of incubation.

As shown in Figure 2, the conidia of *P. microspora* and *P. versicolor* are similar in size, with the width of 4.53–6.65 μm and the length of 18.36–27.88 μm. The length of the top attachment ranged from 9.25 to 26.56 μm, the length of the base attachment ranged from 2.60 to 8.12 μm, and the length of intermediate cells ranged from 11.67 to 17.67 μm. The intermediate cells of conidia of *P. microspore* strain YS26 are monochromatic and almost the same color, light brown. The color of the middle cell is the darkest, and the bottom cell’s color is the lightest. The top and base cells are transparent and colorless, and the shape is fusiform or spindle. There are 2–4 appendages attached to the top (mostly is 3), and one attachment to the base. Furthermore, the germination rate of conidia was similar between glass slides and hard plastic (Figure 2). The conidia began to germinate on both glass slide and hard plastic after 10 h of incubation. It was found that the basal cell of the conidia of *P. microspore* strain YS26 expanded first, and the germ tube grew from the vertical direction of the basal cell or the side of the penultimate cell of the base, which was different from the conidia germination of *P. versicolor* strain XJ27 (Figure 1 and Figure 2).

### 2.2. Bayberry–Pestalotiopsis Interactions

#### 2.2.1. Symptoms Development

**Figure 3:**
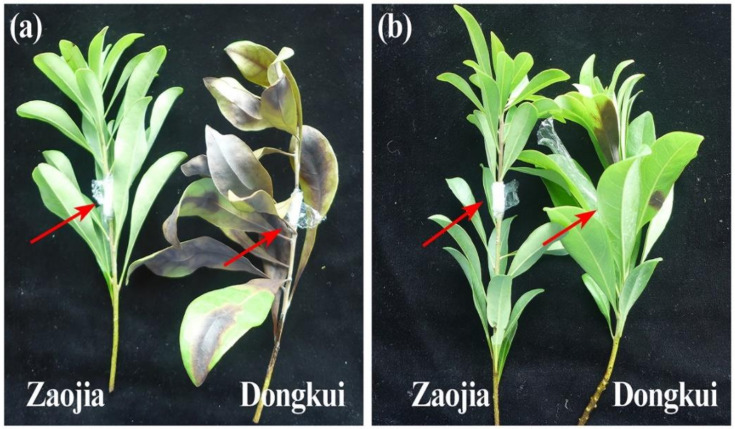
Symptom on the resistant (left, Zaojia) and susceptible (right, Dongkui) cultivar 14 d after inoculation of conidia suspension of *P. versicolor* strain XJ27 (**a**) and *P. microspore* strain YS26 (**b**). The red arrow represents the inoculation points.

As shown in Figure 3, there was a difference in disease symptoms between resistant and susceptible varieties after inoculation of *P. versicolor* strain XJ27 and *P. microspore* strain YS26. Indeed, the leaves of the susceptible variety Dongkui gradually turned from green withered to yellow withered and finally fell off after 6 days of pathogen inoculation with an average latent period of 5 days (Table 1). The surface of the twigs gradually expanded from the inoculation point to the outside, from green to brown. The whole twigs gradually dried up and then withered and died, eventually leading to the whole branches’ death. The disease incidence was 100% and the disease index was 100 at 14 days after inoculation with *P. versicolor* strain XJ27, while the disease incidence was 100% and the disease index was 87.7 at 14 d after inoculation with *P. microspore* strain YS26. In contrast, no obvious disease symptom was observed on leaves, twigs and branches of the resistant variety Zaojia within 14 d after inoculation with conidia suspension of *P. versicolor* strain XJ27 and *P. microspore* strain YS26 (Figure 3, Table 1).

#### 2.2.2. Effects on Conidia and Mycelia

**Figure 4:**
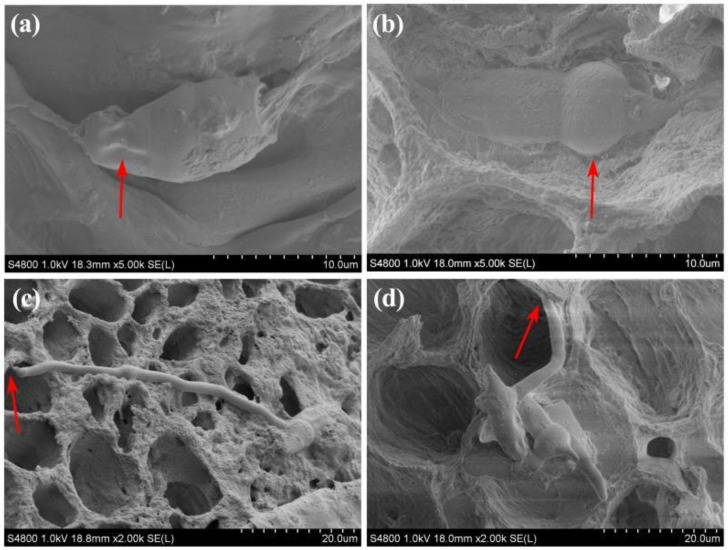
Morphology of conidia and mycelial on branch under cryo-scanning electron microscope after 6 h of pathogen inoculation. (**a**,**b**) conidia on branches of resistant and susceptible cultivar, respectively; (**c**,**d**) mycelial on branches of resistant and susceptible cultivars, respectively. The red arrow in (**a**,**b**) represents the shrunk and expanded cell, respectively, while the red arrow in (**c**,**d**) represents the shrunk and natural mycelium, respectively.

Results from this study indicated that there was an obvious difference between resistant and susceptible varieties in the morphology of conidia and hyphae of *P. versicolor* strain XJ27, which was observed based on the freezing method and cryo-scanning electron microscope (cryo-SEM). As shown in Figure 4a, the conidial morphology of *P. versicolor* strain XJ27 atrophied on the branch of bayberry resistant cultivar Zaojia, while the hyphae shriveled, the ends atrophied and did not continue to extend at 6 h after inoculation (Figure 4c). In contrast, the conidial morphology on the branch of susceptible cultivar Dongkui was full in shape, with an enlarged front end (Figure 4b). The hyphae grew normally, with a tendency to extend into the tissue cells at 6 h of after inoculation (Figure 4d).

#### 2.2.3. Effects on Phloem, Xylem and Medulla

**Figure 5:**
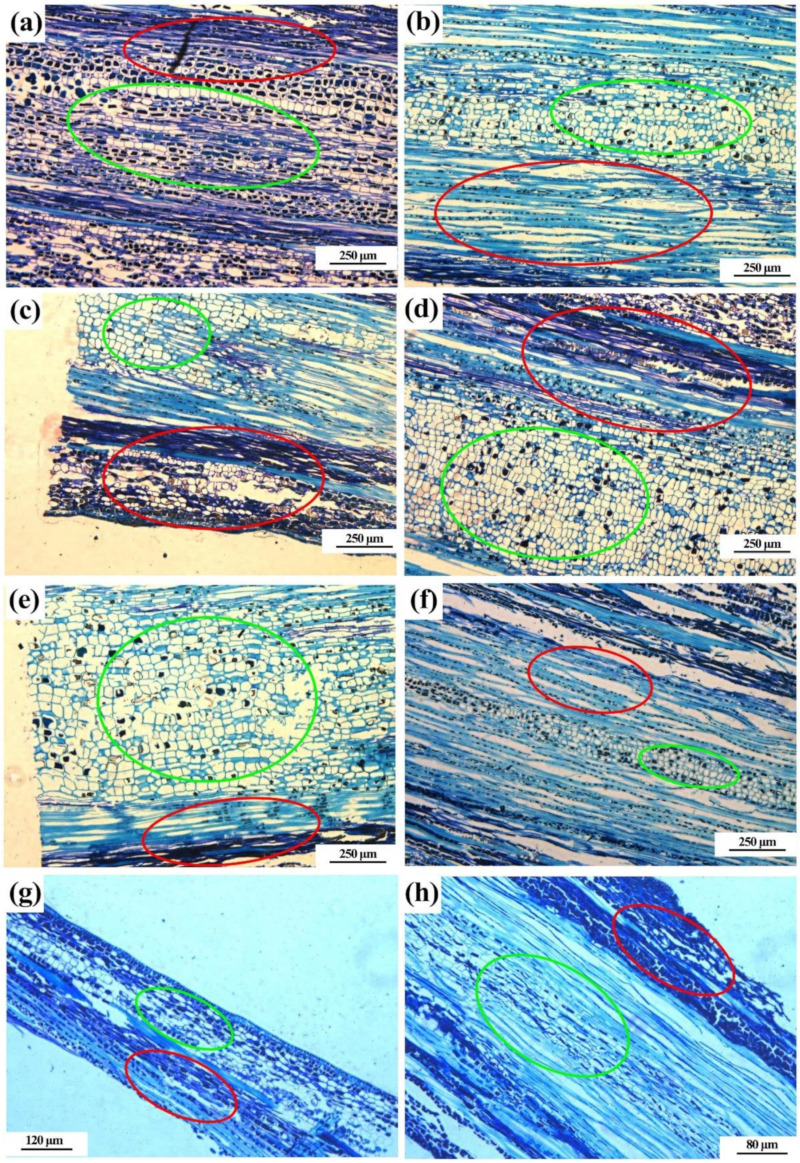
Microscopic observation on the longitudinal section of bayberry resistant (**a**,**c**,**e**,**g**) and susceptible (**b**,**d**,**f**,**h**) cultivars after 0, 6, 48 and 72 h of pathogen infection. The red circle represents phloem and xylem, while the green circle represents medulla.

Results from this study showed that no obvious hyphal infection was found in the phloem, xylem and medulla of resistant and susceptible cultivar after 0, 6, 48 and 72 h of pathogen inoculation through microscopic observation of longitudinal sections of branches. In general, the shape of phloem, xylem and medulla cells was normal in both resistant and susceptible cultivar at four different time points after pathogen inoculation except the medulla cells after 72 h of pathogen inoculation. Indeed, the shape of medulla cells became abnormal in both resistant and susceptible cultivar after 72 h of pathogen inoculation, while a greater abnormality was observed in susceptible cultivar than in resistant cultivar (Figure 5).

**Figure 6:**
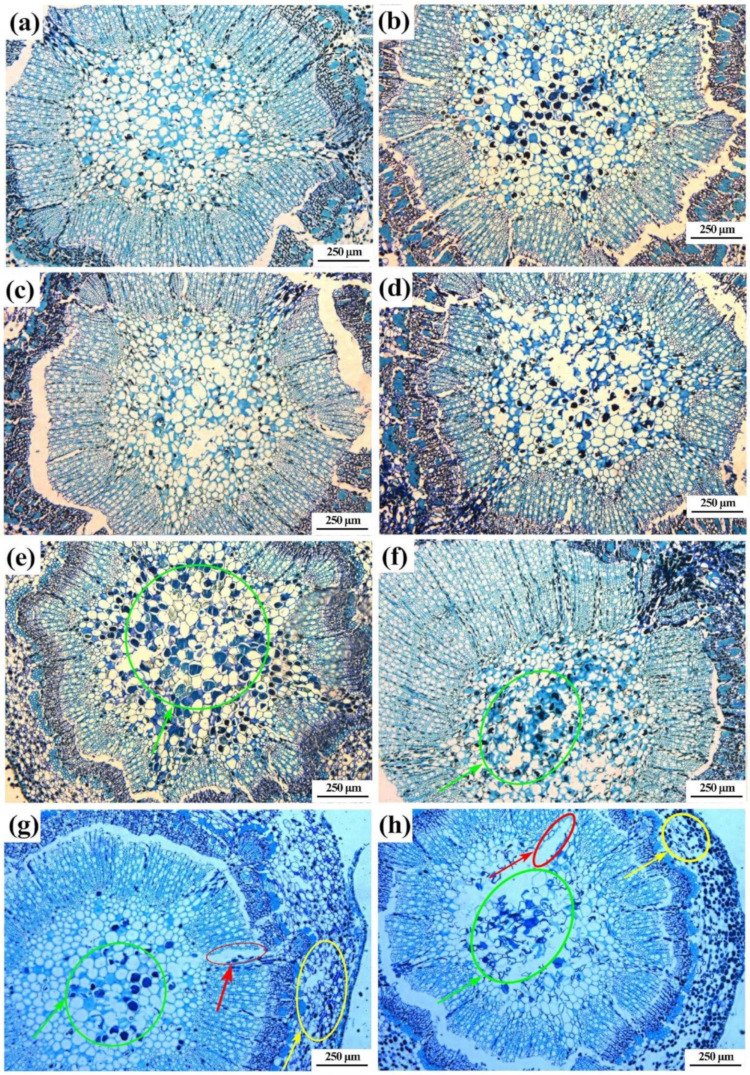
Microscopic observation on the cross-section of bayberry resistant (**a**,**c**,**e**,**g**) and susceptible (**b**,**d**,**f**,**h**) cultivars after 0, 6, 48 and 72 h of pathogen infection. The red and yellow circle represents the xylem and phloem, respectively, while the green circle represents the medulla.

Microscopic observation of cross-section of branches indicated no difference in the phloem of resistant and susceptible cultivar after 0, 6, 48 and 72 h of pathogen inoculation. In addition, the shape of xylem and medulla cells was normal in both resistant and susceptible cultivar at four different time points after pathogen inoculation except the xylem after 72 h of pathogen inoculation and the medulla after 48 and 72 h of pathogen inoculation. In detail, the xylem cells of the susceptible and the resistant cultivar were slightly damaged, while greater damage was observed in susceptible cultivar than in resistant cultivar after 72 h of pathogen inoculation. Furthermore, pathogens infection caused damage to the medulla in susceptible cultivar, but not in resistant cultivar after 48 and 72 h of pathogen inoculation. Indeed, compared to the normal cells in resistant cultivar, the shape of medulla cells became abnormal in susceptible cultivar after 48 and 72 h of pathogen inoculation, while the latter shows greater damage to medulla cells than the former (Figure 6).

### 2.3. Gibberellin Genes Relative Expression

#### 2.3.1. In Leaves

**Figure 7:**
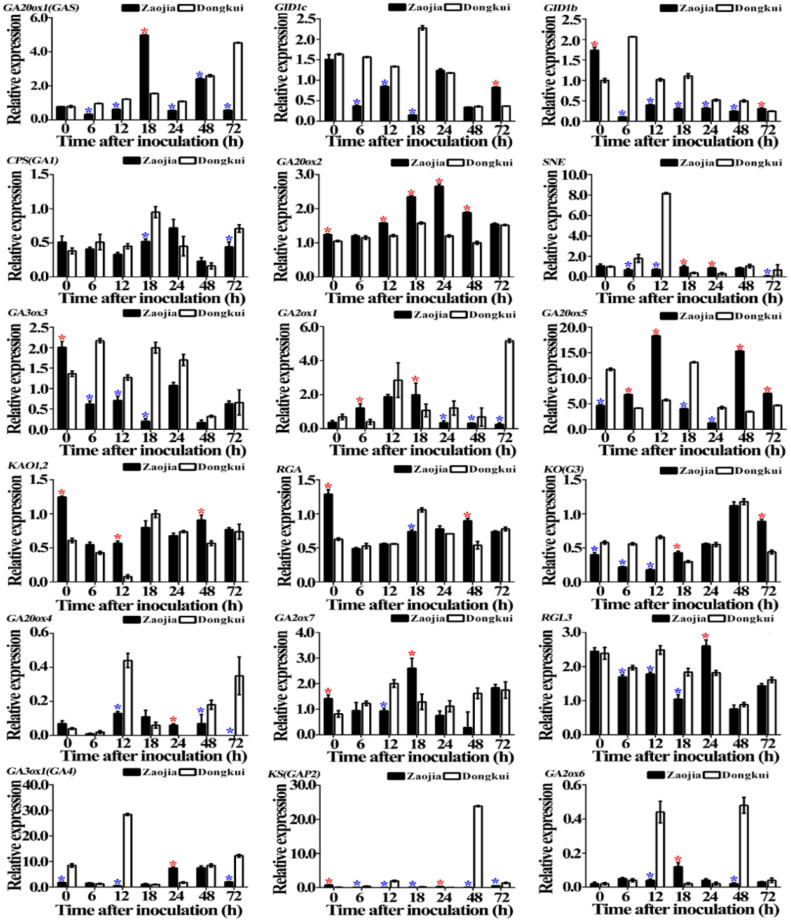
Relative expression of gibberellin signaling pathway-related genes in leaves of resistant (Zaojia) and susceptible (Dongkui) bayberry cultivars following the infection of twig blight pathogen. Data were analyzed by one-way analysis of variance (ANOVA) with least significant difference (LSD) posthoc test. These values are the mean of three repetitions ± standard error of the means. The asterisk (*) in red and blue over bars represent the significant (*p* < 0.05) upregulation and downregulation, respectively, in the expression of one gene in resistant cultivar relative to the susceptible cultivar. *MrUBQ1* was used as the internal reference gene.

Results of quantitative real-time PCR showed that the relative expression of gibberellin signaling pathway-related genes in Table 2 was significantly either increased or reduced or unchanged in leaves of resistant and susceptible bayberry cultivars with the increase of the time after pathogen inoculation. In general, the relative expression of gibberellin signaling pathway-related genes was related to infection time, the type of genes and varieties of bayberry. At 0 h after pathogen inoculation, there was a significant (*p* < 0.05) change in expression of 10 genes in leaves between resistant and susceptible bayberry cultivars, while no significant change was observed in the expression of other eight genes between resistant and susceptible bayberry cultivars. Indeed, the relative expression of seven genes (*GID1b*, *GA20ox2, GA3ox3, KAO1,2, RGA, GA2ox7* and *KS(GAP2)* in resistant cultivars is 1.74-, 1.18-, 1.48-, 2.05-, 2.05-, 1.73- and 7.30-fold, respectively, higher than that of susceptible cultivars, the relative expression of three genes (*GA20ox5, KO(G3), and GA3ox1(GA4)*) in susceptible cultivars is 2.50-, 1.45- and 4.63-fold, respectively, higher than that of resistant cultivars (Figure 7).

However, at 72 h after pathogen inoculation, there was a significant (*p* < 0.05) change in expression of 11 genes in leaves between resistant and susceptible bayberry cultivars, while no significant difference was observed in the expression of other seven genes between resistant and susceptible bayberry cultivars. Indeed, the relative expression of four genes (*GID1c, GID1b, GA20ox5* and *KO(G3)*) in resistant cultivars is 2.24-, 1.24-, 1.50- and 2.02-fold, respectively, higher than that of susceptible cultivars, the relative expression of seven genes (*GA20ox1(GAS), CPS(GA1), SNE, GA2ox1, GA20ox4, GA3ox1(GA4)* and *KS(GAP2)*) in susceptible cultivars is 7.95-,1.61-, 7.44-, 20.64-, 35.00-, 6.18- and 2.50-fold, respectively, higher than that of resistant cultivars (Figure 7).

#### 2.3.2. In Stems

**Figure 8:**
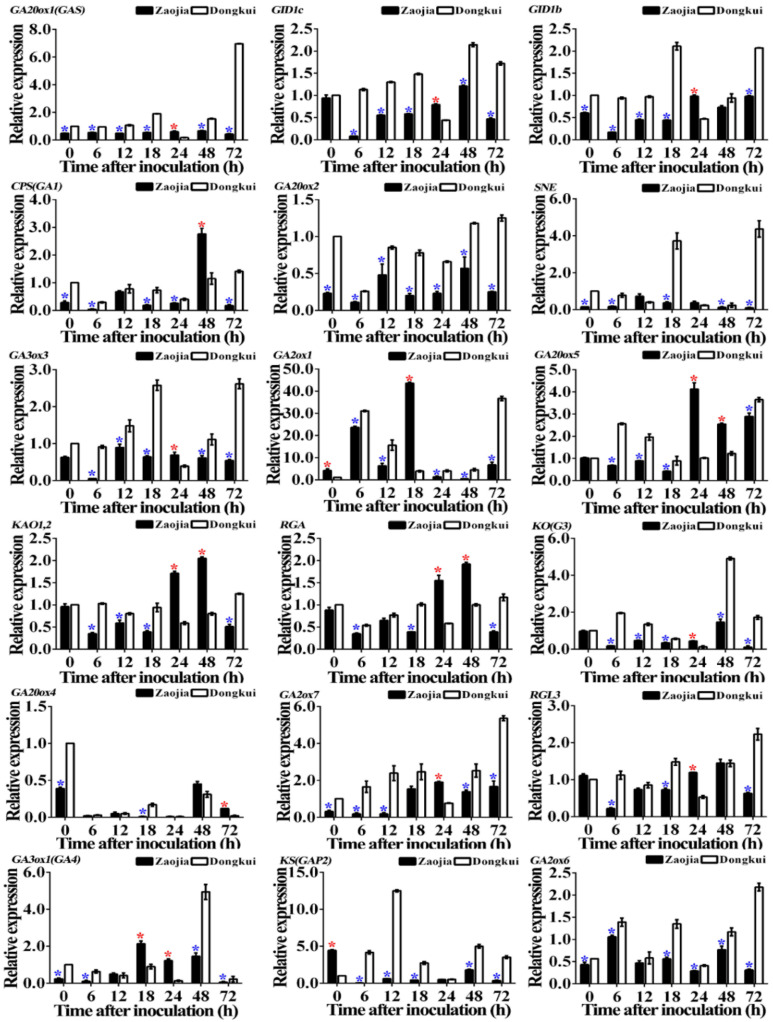
Relative expression of gibberellin signaling pathway-related genes in stems of resistant (Zaojia) and susceptible (Dongkui) bayberry cultivars following the infection of twig blight pathogen. Data were analyzed by one-way analysis of variance (ANOVA) with least significant difference (LSD) posthoc test. These values are the mean of three repetitions ± standard error of the means. The asterisk (*) in red and blue over bars represent the significant (*p* < 0.05) upregulation and downregulation, respectively, in the expression of one gene in resistant cultivar relative to the susceptible cultivar. *MrUBQ1* was used as the internal reference gene.

Results of quantitative real-time PCR showed that the relative expression of gibberellin signaling pathway-related genes in Table 2 was significantly either increased or reduced or unchanged in stems of resistant and susceptible bayberry cultivars with the increase of the time after pathogen inoculation. In general, the relative expression of gibberellin signaling pathway-related genes was related to infection time, the type of genes and varieties of bayberry. At 0 h after pathogen inoculation, there was a significant (*p* < 0.05) change in expression of 11 genes in stems between resistant and susceptible bayberry cultivars, while no significant difference was observed in the expression of the other seven genes between resistant and susceptible bayberry cultivars. Indeed, the relative expression of two genes (*GA2ox1* and *KS(GAP2)*) in resistant cultivars is 4.20 and 4.43, respectively, higher than that of susceptible cultivars, the relative expression of nine genes (*GA20ox1(GAS), GID1b, CPS(GA1), GA20ox2, SNE, GA20ox4, GA2ox7, GA3ox1(GA4)* and *GA2ox6*) in susceptible cultivars is 2.04, 1.67, 3.57, 4.35, 6.67, 2.56, 3.13, 4.17 and 2.33, respectively, higher than that of resistant cultivars (Figure 8).

However, at 72 h after pathogen inoculation, a significant (*p* < 0.05) change in expression of the 18 genes in stems between resistant and susceptible bayberry cultivars. Indeed, the relative expression of *GA20ox4* in resistant cultivars is 6.00-fold higher than that of susceptible cultivars. However, the relative expression of 17 genes (*GA20ox1(GAS), GID1c, GID1b, CPS(GA1), GA20ox2, SNE, GA3ox3, GA2ox1, GA20ox5, KAO1,2, RGA, KO(G3), GA2ox7, RGL3, GA3ox1(GA4), KS(GAP2*) and *GA2ox6*) in susceptible cultivars is 15.82-, 3.66-, 2.11-, 8.29-, 5.00-, 48.55-, 4.85-, 5.42-,1.26-, 2.45-, 3.00-, 15.64-, 3.21-, 3.54-, 3.67-, 10.06- and 7.03-fold, respectively, higher than that of resistant cultivars (Figure 8).

## 3. Discussion

At present, it is generally believed that bayberry twig blight is caused by the two species *P. versicolor* and *P. microspora*, while microscopic observation showed that the conidia of the two species were different in morphology, germination rate and germination mode. Results from this study indicated that the former germinated earlier on glass slides than the latter, which may be the main cause for the fact that *P. versicolor* is the main pathogen of bayberry twig blight.

Cryo-scanning electron microscopic observation indicated that the conidia of *P. versicolor* could be adsorbed on the branch surface of both resistant and susceptible bayberry cultivars, but the morphology of conidia and hyphae were significantly different. The germination of conidia and the growth of hyphae were inhibited in the resistant variety. Similar to the results of this study, the infection ability and mycelial growth rate of *Fusarium* wilt in the resistant varieties were significantly lower than those of the susceptible varieties of cucumber, melon and watermelon [26,27,28]. Furthermore, the resistant lettuce varieties were significantly better than the susceptible varieties in limiting the invasion of *Verticillium dahliae* and mycelial growth ability. However, the pathogen could colonize and infect the resistant varieties [29]. In addition, studies have found that the resistance of resistant varieties to the invasion of pathogens may be mainly due to the thickening of cell wall, accompanied by the formation of structures such as papilla, invading body, wall covering, brown matter and corking of cortical parenchyma cells [30,31].

Microscopic observation of a cross-section of bayberry branches showed that the xylem and medulla cells were intact or slightly damaged in resistant variety, but were obviously damaged in susceptible variety. Similar to the result of this study, some other diseases have also been reported to cause severe damage to the xylem of plant vascular bundles, leading to internal discoloration of vascular bundles and necrosis of terminal tender leaves. For example, the mulberry bacterial wilt was caused by *Ralstonia solanacearum* and *Enterobacter cloacae* [32], while grape pierce disease was caused by *Xylella fastidiosa* [33]. This suggested that the stem withering of bayberry infected with twig blight might be caused by the invasion of *Pestalotiopsis* pathogens toxin into xylem and medulla, killing cells, destroying the internal structure and causing vessel blockage.

In agreement with previous reports, our results from this study revealed that gibberellin signaling pathway-related genes may play an important role in the response of bayberry to the twig blight pathogen’s infection. It has been well shown that jasmonates and salicylic acid activate different pathogenesis-related proteins to regulate plant resistance [22,23]. However, recent studies have shown that, in addition to the widely known jasmonates and salicylic acid, other hormones, especially gibberellin, also play an important role in regulating plant disease resistance, or participate in the interaction between plant host and pathogen by affecting salicylic acid and jasmonates pathways [19,24].

This study indicated that it seems to be very complex for the relative expression of the 18 selected genes involved in the gibberellin signaling pathway, which depend on infection time, the type of genes, and varieties of bayberry. There was a 10.0% increase in the number of differentially expressed genes in bayberry leaves between resistant and susceptible varieties at 72 h after pathogen inoculation compared to that at 0 h after pathogen inoculation, however, there was a 63.6% increase in the number of differentially expressed genes in bayberry stems between resistant and susceptible varieties at 72 h after pathogen inoculation compared to that at 0 h after pathogen inoculation. Furthermore, there was a 63.6% increase in the number of differentially expressed genes in bayberry stems between resistant and susceptible varieties at 72 h after pathogen inoculation compared that in bayberry leaves, this may explain the fact that the xylem and medulla of bayberry was damaged by the pathogen, resulting in systematic twig blight disease.

Gibberellins are a large class of tetracyclic, diterpenoid plant hormones, which has been reported to play fundamental roles in plant growth and development. In recent years, gibberellins are also increasingly implicated in plant responses to pathogen attack, although our understanding of the underlying mechanisms is still limited [24]. However, GA2ox, GA3ox, and GA20ox have been known to be three key enzymes in gibberellins biosynthesis. [20]. In agreement with the results from other researchers, the result of our study indicated that there was a significant change in expression of all eight genes belong to the three groups between resistant and susceptible varieties in stems at 72 h after pathogen inoculation.

It is well known that gibberellin signaling pathway-related genes were highly associated with the formation of plant light morphology by regulating the gibberellin content in plants. Mutations of these genes can also lead to gibberellin deficiency, resulting in extreme plant dwarfing or abortion; such as rice d35 mutants [34] and pea na mutants [35]. Therefore, it can be inferred that compared to the susceptible cultivar Dongkui, the resistance of resistant cultivar Zaojia to twig blight disease may be mainly due to its characteristics of the dwarf tree and short fruit maturity.

Interestingly, the dwarf role of gibberellin in the resistance of bayberry to fungal infection can be justified by the differential expression of the gibberellin receptor encoding genes *GID1* (gibberellin insensitive dwarf1). Indeed, this study indicated that there was a significant change in expression of *GID1b* and *GID1c* between resistant and susceptible varieties in both leaves and stems at 72 h after pathogen inoculation. In agreement with the result of this study, it is concluded that GID1 is a soluble receptor mediating gibberellin signaling in rice. More recently, three GID1 homologs, GID1a, b, and c in *Arabidopsis thaliana* functioned by binding to gibberellin and interact with the five DELLA proteins [36].

On the other hand, the role of gibberellins in plant growth promotion and disease resistance may be, at least partially, due to the induced degradation of the DELLA proteins, which are a class of nuclear growth-repressing proteins and function as plant growth repressors [25]. As we expected, there was a significant change in expression of their DELLAs encoding genes *SNE*, *RGA* and *RGL3* between resistant and susceptible varieties in stems at 72 h after pathogen inoculation. In agreement with the result of this study, De Vleesschauwer et al. (2016) reveal a prominent role of the DELLA protein in the resistance against the rice pathogens *Magnaporthe oryzae* and *Xanthomonas oryzae* pv. *oryzae* [24]. Overall, these results indicated that the gibberellin may play an important role in the process of resistance induction of bayberry.

## 4. Materials and Methods

### 4.1. Plant Material

The bayberry-resistant cultivar Zaojia and susceptible cultivar Dongkui were used in this study, which was collected from Lanxi County, Zhejiang Province, China. The two bayberry cultivars were currently widely planted in Zhejiang province, China, while Zaojia was bred by the Institute of Horticulture, Zhejiang Academy of Agricultural Sciences, China [18].

### 4.2. Fungal Strains

*P. versicolor* strain XJ27 and *P. microspore* strain YS26, the two fungal pathogens of bayberry twig blight, were isolated and identified in our previous studies by Horticulture Institute of Zhejiang Academy of Agricultural Sciences. After routine culture on PDA at 25 °C for 7 days, the fungal pathogen was stored in the refrigerator at 4 °C for use [3]. The inoculum of conidia was prepared by culturing the pathogen on PDA at 25 °C in an incubator for 5 days, and then putting in 12 h light/12 h dark for 5 days to induce conidia to produce.

### 4.3. Conidia Germination and Morphology

Following the induction as described above, the produced conidia were collected by washing them gently with sterile water, filtering them with four layers of gauze, and counting them with a blood cell counting plate, while its concentration was adjusted to 1 × 10^5^ conidia mL^−1^ using sterile water. As the conidia are the main infected organs of the pathogen, the germination and morphology of conidia was determined under the microscope (Olympus BX43) every two hours for 20 h by dropping 4 drops of 1 μL conidia suspension on sterile glass slide and hard plastic slide, and then cultured in the dark in an incubator with a temperature of 28 °C and a humidity of 75–85%.

### 4.4. Bayberry–Pestalotiopsis Interactions

#### 4.4.1. Inoculation and Sampling

The branches of resistant variety Zaojia and susceptible variety Dongkui were inoculated with *P. versicolor* strain XJ27 and *P. microspore* strain YS26 based on the method Ren et al. [3] with minor modification. In brief, we first select the 1-year-old detached branches with a diameter of 0.6–0.9 cm, preculture in 250 mL tap water for 3 days. The inoculation was carried out by carefully pulling out a leaf 5 cm away from the top of the branch, and then putting 20 μL conidia suspension on the leaf scar, while the inoculation point was moisturized using absorbed water cotton and then enclosed using the fresh-keeping film gently. The control was inoculated with sterilized water. All branches were cultured in a 25 °C incubator with saturated humidity at a constant temperature. The experiment was conducted in a completely randomized design with six replicates of each treatment, while each replicate contains one branch. All experiments were repeated twice.

#### 4.4.2. Symptoms Development and Disease Investigation

The disease symptom was investigated daily until 14 days after inoculation of pathogen. The plant samples were taken from the resistant and susceptible varieties at 0, 6, 12, 18, 24, 48 and 72 h after inoculation of strain XJ27 of *P. versicolor*, which are more virulent to bayberry than that of *P. microspore* strain YS26. For histological observation and gibberellin genes relative expression, the shoots were sampled at 1 cm above and below the inoculation point, while the leaves were sampled from the above and below the inoculation point were sampled. The disease was investigated every day to determine the average latent period (days from inoculation to symptom appearance) and disease incidence. The disease index was calculated according to the following formula.

Disease index = ∑ (number of diseased branch × representative value of the grade) × 100/(total number of investigation branch × representative value of the highest grade)(1)

The degree of twig blight was divided into six grades (0, 1, 3, 5, 7 and 9) based on the percentage (0, <10%, 10–25%, 25–50%, 50–75% and 75–100%) of dry or falling leaves in relation to the total leaves of one branch, respectively.

#### 4.4.3. Effects on Conidia and Mycelia

In order to provide a theoretical basis for an understanding of the infection of twig blight pathogen to bayberry, the infection organs of twig blight pathogen were histologically observed in resistant and susceptible varieties of bayberry, which was carried out by removing the surface water, quickly freezing in liquid nitrogen, and then putting the fresh sample into the cryoscope (S-4800 field emission scanning electron microscope).

#### 4.4.4. Effects on Phloem, Xylem and Medulla

The response of bayberry to twig blight pathogen was examined by histologically observing the fungal pathogen’s infection process in resistant and susceptible varieties. In brief, the paraffin-embedded sections of the bayberry branches samples were prepared as described in our previous studies following the fixation with Formaldehyde-acetic acid-ethanol Fixative (FAA fixative) [37]. The infection and destruction of the phloem, xylem and medulla of branches were determined under the microscopic observation of cross and longitudinal section after 0, 48 and 72 h of pathogen inoculation.

### 4.5. Gibberellin Genes Relative Expression

#### 4.5.1. Genes Selection and Primers Design

In order to examine the response of bayberry to twig blight infection at the molecular level, the gibberellin signaling pathway-related genes were obtained from the previous publications [36,37,38,39] and the genome database of bayberry (bayberrybase.cn). As shown in Table 2, the 18 representative genes involved in the gibberellin-signaling pathway were included in this study. Furthermore, each gene-specific primer was successfully designed using Primer Premier 5.0 software and Primer 3.0 online (http://bioinfo.ut.ee/primer3-0.4.0/, accessed on 21 August 2016) based on the nucleotide sequence of each gene, which was justified by producing a single band with the size of from 85 bp to 150 bp. Table 2 listed the information of the primer sequence and the putative function of each gene.

#### 4.5.2. Relative Expression Analyses

In order to examine the differential expression of gibberellin signaling pathway-related genes in resistant and susceptible varieties of bayberry, RNA samples from leaves and branches of bayberry were extracted with Mini BEST Plant RNA Extraction Kit, which purchased from TaKaRa Company (Dalian, China). Following precooling with liquid nitrogen, the plant tissue samples were quickly ground to powder in the mortar, which was wrapped with aluminum foil and then placed in the oven at 160 °C for 6 h to ensure the removal of RNase. RNA degradation was further ruled out by gel electrophoresis. cDNA was synthesized by using the PrimeScript™ RT reagent Kit with gRNA Eraser (Perfect Real Time, TaKaRa Company, Dalian, China). The quantitative real-time PCR reaction was carried out as described in our previous study [18] in LightCycler^®^ (Roche Diagnostics) amplification instrument using SYBR^®^ Premix DimerEraser™ (Perfect Real Time, TaKaRa Company). The relative expression of each gene was analyzed using the 2^−ΔΔCt^ method with *MrUBQ1* as the internal standard. The fold change was calculated by comparing the relative expression level of each gene in resistant variety to susceptible variety. Each result represents the average of three independent determinations. This experiment was repeated twice.

### 4.6. Statistics Analysis

The software STATGRAPHICS Plus, version 4.0 (Copyright Manugistics Inc., Rockville, Md., USA) was used to perform the statistical analysis. Data were analyzed by one-way analysis of variance (ANOVA) with the least significant difference (LSD) posthoc test. *p* < 0.05 was considered as statistical significance.

## 5. Conclusions

In conclusion, results from this study revealed that there was a differential interaction between twig blight pathogen and bayberry resistant or susceptible cultivars, while the gibberellin signaling pathway-related genes play an important role in the interaction between bayberry and twig blight and may be able to be used as molecular markers for resistance breeding of bayberry. Overall, the result of this study helps us understand the infection process of twig blight pathogen and provided a clue for us to understand the resistance mechanism of resistant cultivar to the fungal infection in bayberry.

## Figures and Tables

**Table 1:** Pathogenicity of *P. versicolor* strain XJ27 and *P. microspore* strain YS26 on two bayberry cultivars.

Cultivars	Latent Period (d)		Disease Incidence (%)		Disease Index	
	XJ27	YS26	XJ27	YS26	XJ27	YS26
Dongkui	5	5	100 a	100 a	100 a	87.7 a
Zaojia	/	/	0 b	0 b	0 b	0 b

Disease incidence and index was investigated after 14 d of pathogen inoculation. Data were analyzed by one-way analysis of variance (ANOVA) with least significant difference (LSD) posthoc test. The different letters in the same column show significant difference at 5% level.

**Table 2:** Primers of quantitative real-time PCR used in this study.

Primer Name	Gene Name	Nucleotide Sequence (5′–3′)	Putative Function of Target Gene	PCR Product
015554-F	*GA20ox1(GAS)*	CACGATCCCCACGATTGACA	2OG-Fe(II) oxygenase superfamily	144 bp
015554-R		GCCCTATGCTCCACGCTATT		
007568-F	*GID1c-*	CGTCGCCCTGATGGTACTTT	Gibberellin receptor GID1C GN	88 bp
007568-R		AAACTCCATCCACCGGCTTT		
013954-F	*GID1b*	AAATGAGGCTCAATGGGGCA	Gibberellin receptor GID1B GN	142 bp
013954-R		GCGGCGACAAAAAGTGTCAT		
017149-F	*CPS(GA1)*	ATGACACGGCATGGGTTTCT	Ent-copalyl diphosphate synthase	117 bp
017149-R		TATCGCCCCATGAACCATCG		
016673-F	*GA20ox2*	CGTGCGGTGAGGAAATCTCT	Gibberellin 20 oxidase 1 GN	123 bp
016673-R		AGCTGGCCATCTGAAGTCTG		
010912-F	*SNE*	AGCGGCTCTACATGGTATGC	F-box protein SNE GN	150 bp
010912-R		ACGCATCACCGAGTCTTCTC		
010100-F	*GA3ox3*	ACCGGGCGATGTGAAGATTT	Gibberellin 3-beta-dioxygenase 1 GN	85 bp
010100-R		TTCCTTCCATGTTACCGGGC		
019537-F	*GA2ox1*	AATGGGAGGTTCCAAAGCGT	Gibberellin 2-beta-dioxygenase 1 GN	98 bp
019537-R		TTCTCACTCAAAGGCGGTCC		
003769-F	*GA20ox5*	AGTCTCATTCGCGCTGCATA	Gibberellin 20 oxidase 2 OS	127 bp
003769-R		AATAACGGTCTGCATGGGCA		
012730-F	*KAO1,2*	TTTCCTACAGCAGCACCCAG	Ent-kaurenoic acid oxidase 2 GN	99 bp
012730-R		TTCAGCGTCAACCCACTCTG		
011275-F	*RGA*	TTTCCTACAGCAGCACCCAG	DELLA protein GAIP-B	99 bp
011275-R		TTCAGCGTCAACCCACTCTG		
002577-F	*KO(G3)*	GCTTTGGGACATGACCTGGA	Ent-kaurene oxidase	103 bp
002577-R		CACCTTCCATTACGTCGGCT		
019330-F	*GA20ox4*	TTCGAACAGACAGGAGTGGC	Gibberellin 20 oxidase 2 OS	101 bp
019330-R		CGATCGACTCCCAAGCTGAT		
005597-F	*GA2ox7*	TCCTCTGGTCGGAAGCCTTA	gibberellin 2-beta-dioxygenase 8	116 bp
005597-R		TAAGCTTTGAGCCAGGTCGG		
004052-F	*RGL3*	CGGACAACACTGACGCTTTG	DELLA protein GAI	132 bp
004052-R		TATCGAGCATGGACGGTTCG		
001863-F	*GA3ox4*	ATGGGTCTAGCAGCGCATAC	gibberellin 3 oxidase 1	106 bp
001863-R		GAACCATGATCCACCCCGTT		
009378-F	*KS(GAP2)*	CTCTGTGATGGCTCATGGGG	ent-kaurene synthase	110 bp
009378-R		TCACCAACACCCCATTGCTT		
025327-F	*GA2ox6*	CCGGCTCGTCCTTTTGGTTA	Gibberellin 2-beta-dioxygenase 2 GN	140 bp
025327-R		AATTTCGTCGGGTCGTTGGA		
021433-F	*MrUBQ1*	AAGGCGAAGATCCAAGACAA	used as the internal reference	116 bp
021433-R		GTGGAGCGTCGACTCTTTCT		

F: forward primer; R: reverse primer.

## Data Availability

All data supporting the conclusions of this article are included in this article.
